# Association Between Serum PADI‐4 Levels and Disease Activity in Ulcerative Colitis Patients

**DOI:** 10.1155/grp/8211063

**Published:** 2026-04-06

**Authors:** Semiha Şimşek, Elvan Işık, Betül Çiğdem Yortanlı, Cem Çekiç

**Affiliations:** ^1^ Department of Internal Medicine, Konya City Hospital, Konya, Türkiye; ^2^ Department of Gastroenterology, Ege University School of Medicine, Izmir, Türkiye, ege.edu.tr; ^3^ Department of Gastroenterology, Tınaztepe University, Izmir, Türkiye

**Keywords:** disease activity, peptidyl arginine deiminase-4, ulcerative colitis

## Abstract

**Objectives:**

PADI‐4 is a member of the peptidyl arginine deiminase enzyme family. This enzyme is expressed by inflammatory cells and plays an important role in inflammation and immune responses. In this study, we aimed to evaluate serum PADI‐4 levels in patients with ulcerative colitis (UC) and to investigate the relationship between PADI‐4 and disease activity.

**Material and Method:**

Fifty‐one patients with UC and 31 healthy controls were included in this observational study. First, serum PADI‐4 levels in patients with UC and the control group were compared. Subsequently, serum PADI‐4 levels were analyzed in UC patients with active disease and those who are in remission, separately. Finally, the impact of PADI‐4 on predicting disease activity was evaluated by performing correlation analyses between serum PADI‐4 and C‐reactive protein (CRP) in addition to the total Mayo score (TMS) and modified Mayo Endoscopic Subscore (MESS).

**Results:**

Serum mean PADI‐4 levels were found to be significantly higher in the UC group (1317 ± 818 pg/mL) compared to the control group (873 ± 622 pg/mL) (*p*: 0.009). Serum mean PADI‐4 levels were significantly different between the three groups (patients with remission, mild disease, and moderate/severe disease group) (*p*: 0.015) (1076 ± 609, 1073 ± 683, and 1802 ± 943 pg/mL, respectively). In Spearman′s correlation analysis, it was determined that there was a positive and significant relationship between PADI‐4 and CRP levels (*r* = 0.31, *p*: 0.027) in addition to TMS (*r* = 0.419, *p*: 0.02) and MESS (*r* = 0.417, *p*: 0.002). The ideal serum PADI‐4 levels for predicting disease activity were found to be 980 pg/mL.

**Conclusion:**

Our study revealed that serum PADI‐4 levels were higher in patients with UC compared to healthy controls and might be an effective marker to determine disease severity.

## 1. Introduction

Ulcerative colitis (UC) is an inflammatory bowel disease (IBD) caused by an abnormal immune response that begins with the disruption of the intestinal epithelial barrier [1]. Although the exact cause is unknown, genetic factors, environmental factors, intestinal microbiota, and immune system pathologies are thought to play a role in pathogenesis [2]. Determining disease severity is important for both determining treatment options and predicting the prognosis. Although C‐reactive protein (CRP) and fecal calprotectin (FC) are commonly used to determine disease activity in UC, these inflammatory markers, which also have some disadvantages, are not specific to the disease [3]. The Mayo scoring system, which is frequently used to determine clinical and endoscopic disease activity, requires an invasive technique such as colonoscopy [4]. A different, more specific, and less invasive activity marker is needed to determine UC disease activity.

Peptidyl arginine deiminase (PADI) is a group of isoenzymes that catalyze the conversion of arginine to citrulline, called the citrullination or deamination process [5]. At the physiological activity level, PADIs regulate many cell signaling pathways, including differentiation, apoptosis, and gene transcription. Five isoenzymes (PADI 1–4 and 6) exist in mammals. While all PADIs are found in the cell cytoplasm, PADI‐4 is the only isoenzyme that plays a role in histone deimination and is found in the cytoplasm and nucleus [6].

In recent studies, it has been shown that abnormal PADI activity is associated with inflammatory diseases such as rheumatoid arthritis (RA) and neurodegenerative diseases such as Alzheimer′s, multiple sclerosis, Parkinson′s, and several cancer types [7, 8]. The accumulation of citrullinated proteins is thought to be the condition that exacerbates the inflammatory response in immune‐mediated diseases like UC and RA [9, 10]. Particularly, PADI‐4 dysregulation is more common in UC and colorectal cancer [11, 12].

This study planned to investigate the role and effectiveness of serum PADI‐4 level in determining clinical and endoscopic disease activity in UC patients.

## 2. Material and Method

### 2.1. Patient Selection

This study included 51 patients with clinical, endoscopic, radiological, and histopathological diagnoses of UC and laboratory tests and sigmoidoscopy or colonoscopy data within the last 2 weeks who were being followed up in the Gastroenterology Department of Izmir Kâtip Çelebi University Faculty of Medicine Hospital. The control group consisted of 31 healthy individuals.

### 2.2. Exclusion Criteria

Patients who are younger than 18 years of age, have signs of active infection, have chronic renal failure and chronic liver disease, have chronic inflammatory diseases such as rheumatological or collagen tissue disease, have a history of colorectal surgery, have UC with only rectal involvement, and are pregnant and lactating women were not included in the study.

### 2.3. Study Design

Demographic data and disease characteristics of UC patients and healthy controls included in the study were recorded (age, gender, UC localization, treatments received, disease age, disease severity, and endoscopic features). To determine the disease activities of UC patients, outpatient clinic data records, sigmoidoscopy, and colonoscopy examinations of the last 2 weeks were examined. The total Mayo score (TMS) and Mayo Endoscopic Subscore (MESS) of the patients were calculated; accordingly, those with TMS ≤ 2 were designated as in remission, those with TMS 3–5 as having mild–severe disease, and patients with TMS 6–12 as the moderate and severe disease group [12].

Blood samples were taken from healthy controls and UC patients included in the study to measure serum PADI‐4 and CRP levels. First, serum PADI‐4 levels were compared between the UC patient and control groups. Subsequently, serum PADI‐4 levels were compared between patients with different disease severities (remission, mild disease, moderate–severe disease) in the UC group. Then, the effect of demographic data and disease characteristics on serum PADI‐4 levels was determined. Finally, correlation analyses were performed between serum PADI‐4 levels and CRP, TMS, and MESS to investigate the effectiveness of PADI‐4 in determining disease severity.

### 2.4. Evaluation of Serum PADI‐4 Levels

Then, 8–10 mL of venous blood was taken from each subject in the UC patients and the control group, and their serum was separated by centrifuging at 3000 rpm for 15 min under sterile conditions. Serums were stored in clean and dry Eppendorf tubes at −20°C in the deep freezer until the time of use. Hemolyzed and lipemic samples were not included in the study. Patient serum and calibrators were pipetted into antibody‐coated wells. It was incubated for 90 min at 37°C. After incubation, the contents of the plate were spilled. Biotin‐labeled antibody was added to each well, incubated for 1 h at 37°C, and then washed three times with 350 *μ*L of washing solution. Streptavidin‐labeled HRP enzyme was added and incubated for half an hour at 37°C, and then automatic washing was performed five times with 350 *μ*L of washing solution. After adding the substrate for the HRP enzyme, the reaction was terminated using H_2_SO_4_ after incubation at 37°C for 15 min in the dark. Absorbances were read at 450 nm on the ELISA plate reader, and the concentration was calculated according to the standard absorbance curve. For the ELISA method, the Biotek (ELx800, United States) brand semiautomatic ELISA device with the PADI‐4 ELISA kit (catalog no.: E‐EL‐H1322, lot no.: HJNWE2R4SN, Elabscience, United States) was used.

### 2.5. Statistical Analysis

The suitability of the numerical variables included in the study for normal distribution was evaluated with the Shapiro–Wilk test. Numerical variables were described as mean and standard deviation or median and interquartile difference, and categorical variables were described as percentage values. The means of two independent groups were compared by the Mann–Whitney *U* test, and the independent means of more than two groups were compared by the Kruskal–Wallis test and then by post hoc Dunn′s test. The difference between the median of two independent groups was examined with the Mann–Whitney *U* test. The relationship between numerical variables was investigated with the Spearman correlation analysis, and the relationship between categorical variables was investigated with the chi‐square test. The Youden method was used for threshold value analysis. ROC analysis was used to understand the ability of the PADI‐4 level to predict disease activity. The study was conducted at a 95% confidence level (*p* < 0.05 was considered a statistically significant difference).

### 2.6. Ethical Considerations

The study was approved by the Izmir Katip Çelebi University Atatürk Training and Research Hospital Clinical Research Local Ethics Committee (dated 08.13.2020 and Decision Number 63). Informed consent was obtained from all study participants.

## 3. Results

Fifty‐one UC and 31 healthy controls were evaluated in the study. The average age of UC patients was 42.1 ± 13.0 years, and 23 (45.1%) patients were female. In the control group, the average age was 33.2 ± 11.4 years, and 12 (38.7%) were female. It was observed that the average age was statistically lower in the control group (*p*: 0.003). There was no statistical difference between the groups in terms of gender distribution (*p*: 0.571). Demographic and clinical characteristics of the UC patients included in the study are listed in Table 1.

**Table 1:** Demographic and characteristic features of UC patients.

Disease duration, median (IQR)	6	(7)
Disease localization, *n* (%)		
Left colitis	27	(52.9)
Extensive colitis	24	(47.1)
Treatment, *n* (%)		
ASA	31	(60.8)
AZA	5	(9.8)
Biological agent	11	(21.6)
ASA and AZA	3	(5.9)
AZA and biological agent	1	(2)
CRP (mg/dL), mean (SD)	0.88	(2.18)
Mayo Endoscopic Subscore, median (IQR)	1	(2)
MAYO total score, median (IQR)	4	(6)
Disease activity, *n* (%)		
Remission	19	(37.3)
Mild disease	15	(29.4)
Moderate to severe disease	17	(33.3)

Abbreviations: ASA, aminosalicylic acid; AZA, azathioprine; IQR, interquantile range.

**Figure 1:**
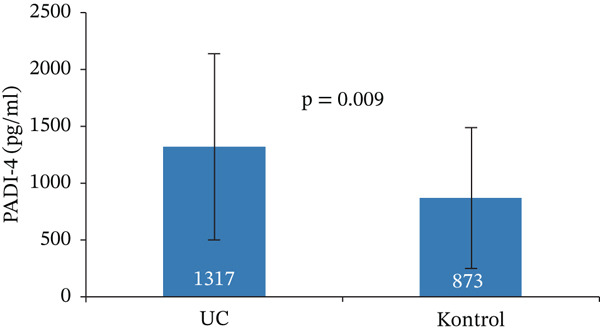
Serum PADI‐4 levels among study groups.

While the mean (SD) level of PADI‐4 in the UC group was 1317 ± 818 pg/mL, the mean (SD) level of PADI‐4 in the control group was 873 ± 622 pg/mL. Serum PADI‐4 levels were found to be statistically significantly higher in the UC group (*p*: 0.009) (Figure 1).

In examining the effects of demographic data and disease characteristics on serum PADI‐4, it was observed that the effects of variables such as age, gender, disease age, disease localization, and different agents used in treatment on serum PADI‐4 levels did not reach statistical significance (Table 2).

**Table 2:** Effect of demographic data and disease characteristics on PADI‐4.

	*n*	PADI‐4 (pg/mL)	*p*
Age (Spearman′s *r*)	51	0.039	0.785
Gender (mean ± SD)			
Female	23	1119 ± 646	0.140
Male	28	1478 ± 915	
Disease duration (Spearman′s *r*)	51	−0.099	0.490
Disease localization (Spearman′s *r*)			
Left colitis	27	1390 ± 710	0.234
Extensive colitis	24	1235 ± 933	
Treatment (mean ± SD)			
ASA	31	1244 ± 900	0.656
AZA	5	1464 ± 766	
Biological agent	11	1313 ± 701	
ASA and AZA	3	1600 ± 647	
AZA and biological agent	1	2039	

Abbreviations: ASA, aminosalicylic acid; AZA, azathioprine; SD, standard deviation.

**Figure 2:**
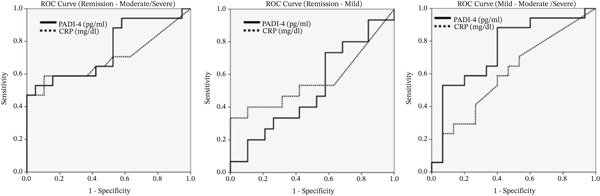
ROC analyses of serum PADI‐4 and CRP levels in predicting disease.

In the correlation analysis between serum PADI‐4 levels and CRP and Mayo Endoscopic Subscore, a positive and statistically significant correlation was detected between CRP (*p*: 0.027), MESS (*p*: 0.002), and TMS (*p*: 0.002) and serum PADI‐4 levels (Table 3). When serum PADI‐4 levels are examined according to disease activity, serum PADI‐4 levels are found to be statistically higher in patients with moderate and severe disease than in patients with mild disease and who are in remission (*p*: 0.015) (Table 4 and Figure 2). Serum PADI‐4 and CRP levels were not found to be significant in determining disease activity between remission and mild disease (*p*: 0.903 and *p*: 0.532, respectively). It was determined that there was a statistical difference between remission and moderate and severe disease, more clearly in serum PADI‐4 levels (*p*: 0.011). A statistical difference was detected between mild and moderate and severe disease only in serum PADI‐4 levels (*p*: 0.015) (Table 5 and Figure 2).

**Table 3:** Correlation analysis between serum PADI‐4 levels and CRP and Mayo Endoscopic Subscore.

	*n*	PADI‐4 (pg/mL)	*p*
CRP (mg/dL) (Spearman′s *r*)	51	0.31	0.027
Mayo Endoscopic Subscore (Spearman′s *r*)	51	0.417	**0.002**
MAYO total score (Spearman′s *r*)	51	0.419	**0.002**

*Note*: Statistically significant correlations were observed between serum PADI‐4 levels and MAYO endoscopic subscore and MAYO total score (Spearman′s correlation, p < 0.05).

**Table 4:** Evaluation of serum PADI‐4 levels according to disease activity.

	*n*	PADI‐4 (pg/mL)	*p*
Disease activity (mean ± SD)			
Remission	19	1076 ± 609	**0.015**
Mild–severe disease	15	1073 ± 683	
Moderate to severe disease	17	1802 ± 943	

*Note*: Statistically significant differences in serum PADI‐4 levels were observed in patients with mild–severe disease (p = 0.015).

**Table 5:** Evaluation of serum PADI‐4 and CRP levels in predicting disease activity.

	AUC	95% CI	*p*
Remission vs. mild			
PADI‐4 (pg/mL)	0.512	0.312–0.712	0.903
CRP (mg/dL)	0.563	0.352–0.775	0.532
Remission vs. moderate to severe			
PADI‐4 (pg/mL)	0.749	0.583–0.915	**0.011**
CRP (mg/dL)	0.695	0.509–0.881	**0.046**
Mild vs. moderate to severe			
PADI‐4 (pg/mL)	0.753	0.578–0.928	**0.015**
CRP (mg/dL)	0.602	0.403–0.800	0.326

*Note*: Statistically significant differences in PADI‐4 and CRP levels were observed in the following comparisons: PADI‐4 and CRP in patients with remission versus moderate to severe disease (p = 0.011 and p = 0.046, respectively) and PADI‐4 in patients with mild versus moderate to severe disease (p = 0.015).

The Youden method was used to determine the most appropriate serum PADI‐4 level in evaluating disease activity, and the most ideal serum PADI‐4 level in predicting activity was determined to be 980 pg/mL. When patients with serum PADI‐4 levels below and above 980 pg/mL were evaluated as two separate groups, it was found that TMS and MESS were statistically significantly higher in the group with PADI‐4 levels above the cut‐off value (*p*: 0.017 and *p*: 0.011, respectively). Detailed statistical data regarding the optimal Youden cut‐off values are presented in Table 6. CRP levels did not reach statistical significance between groups (*p*: 0.058). The PADI‐4 cut‐off level (980 pg/mL) determined in the evaluation between remission, mild, and moderate–severe patient groups was found to be statistically significant (*p*: 0.013).

**Table 6:** Diagnostic performance of PADI‐4 for determining disease severity according to the optimal Youden cut‐off.

*P* *A* *D* *I* − 4 ≥ 980 pg/mL	Remission vs. mild	Remission vs. moderate/severe	Mild vs. moderate/severe
Sensitivity (95% CI)	0.400 (0.163–0.677)	0.882 (0.636–0.985)	0.882 (0.636–0.985)
Specificity (95% CI)	0.474 (0.245–0.711)	0.474 (0.245–0.711)	0.600 (0.323–0.837)
PPV (95% CI)	0.375 (0.220–0.560)	0.600 (0.486–0.704)	0.714 (0.568–0.826)
NPV (95% CI)	0.500 (0.348–0.652)	0.818 (0.530–0.947)	0.818 (0.535–0.946)
Accuracy (95% CI)	0.441 (0.272–0.621)	0.667 (0.490–0.814)	0.750 (0.566–0.885)

## 4. Discussion

UC is a lifelong chronic inflammatory disease of the colon of unknown cause. A complex interaction of genetic, environmental, and immunological factors is responsible for the pathogenesis of the disease [13, 14]. Further understanding of these mechanisms involved in pathogenesis in recent years, especially the identification of cytokines having a role in disease development, has also affected treatment strategies. The aim of treatment in IBD is not only to control the symptoms of the patient but also to prevent uncontrolled inflammation and to ensure mucosal healing [15]. Endoscopic procedures used to evaluate mucosal healing are invasive and expensive. For this reason, many easier and noninvasive biochemical and serological markers have been studied to determine disease severity [16]. To date, the most commonly used inflammatory markers to determine disease activity and severity of inflammation in IBD are CRP and FC [17]. There are studies supporting that CRP is a marker having a correlation with the clinical activity of the disease and the degree of inflammation in IBD [18]. However, negative factors such as variation in CRP gene polymorphism causing interpersonal differences, mucosal confinement in UC, and increased serum levels in infectious and inflammatory processes limit the use of CRP in the follow‐up of IBD. FC is also affected by factors such as colon polyps, colon diverticular disease, colorectal neoplasia, and nonsteroidal anti‐inflammatory drug use; this is seen as a disadvantage in clinical practice [19].

PADI is a family of enzymes with different isoenzyme subgroups that play a role in converting peptidyl arginine to peptidyl citrulline through citrullination. At physiological levels, it plays a role in cell differentiation, nerve cell development, apoptosis, embryonic differentiation, and gene transcription [6]. Studies on the PADI family and related diseases were conducted in the early 2000s. However, in recent years, there has been an increase in studies investigating the role of PADI and its enzyme subgroups in the pathogenesis of neurological and oncological diseases, especially inflammatory processes such as rheumatic diseases [20–22]. One reason why PADI‐4 was chosen as the research subject in this study is that the formation of neutrophil extracellular traps (NETs), a protein and chromatin complex secreted by activated neutrophils, is upregulated by PADI‐4. NETs are thought to have both protective and pathogenic effects on IBD intestinal inflammation [23].

In our study, serum PADI‐4 levels were found to be statistically significantly higher in the UC patient group compared to the control group (*p*: 0.009). Although there is no study investigating serum PADI‐4 levels in UC patients, Dinallo et al. showed in their study that PADI‐4 levels were increased in inflamed mucosa [24].

In the correlation analysis performed between serum PADI‐4 levels and CRP and Mayo Endoscopic Subscore in our study, it was seen that there was a positive and statistically significant correlation between CRP, MESS, TMS, and serum PADI‐4 levels. In the ROC analysis performed between groups with different disease activity to compare the power of CRP and PADI‐4 in determining inflammation, it was observed that serum PADI‐4 had a wider AUC compared to CRP, especially in patient groups with higher inflammatory burden. When serum PADI‐4 levels are examined according to disease activity determined based on TMS, serum PADI‐4 levels are found to be statistically higher in patients with moderate and severe disease than in patients with mild disease and those in remission (*p*: 0.015). When the literature was examined, no study was found investigating the relationship between serum PADI levels and disease activation in UC patients. However, Leppkes et al. showed in their study that PADI‐4 levels were high in inflamed mucosa in UC and that there was a parallelism between mucosal damage and PADI‐4 activity [25].

As highlighted in the STRIDE‐II study, which redefined therapeutic targets in IBD, mucosal healing and endoscopic remission remain the most important determinants for predicting disease course and complications in UC [15]. In our study, serum PADI‐4 levels were found to correlate positively with the Mucosal Endoscopic Severity Score (MESS), and the PADI‐4 cut‐off value (980 pg/mL) was also associated with statistically significant differences in MESS (*p* = 0.011).

There are some limitations in our study. The lack of a statistical difference in age between the patient and control groups; the fact that no other inflammatory markers, such as FC or IL‐6, were evaluated other than CRP; and the lack of homogeneous distribution among the treatment subgroups can be stated as the shortcomings of our study. On the other hand, the determination of mucosal expression of PADI‐4 in biopsy samples, as well as serum PADI‐4 levels, can be shown as a factor that will increase the power of the study.

In conclusion, in light of these data, it was concluded that serum PADI‐4 levels may be a helpful method for determining disease activity in patients with UC; however, although PADI‐4 appears to show a stronger association with inflammatory activity than CRP, it may not be sufficient on its own and should be considered as a supportive tool, especially in patients with severe inflammatory activity.

**Nomenclature**
CRP: C‐reactive protein
FC: fecal calprotectin
IBD: inflammatory bowel disease
MESS: modified Mayo Endoscopic Subscore
NETs: neutrophil extracellular traps
PADI: peptidyl arginine deiminase
RA: rheumatoid arthritis
TMS: total Mayo score
UC: ulcerative colitis

## Funding

No funding was received for this manuscript.

## Conflicts of Interest

The authors declare no conflicts of interest.

## Data Availability

The data that support the findings of this study are available upon request from the corresponding author. The data are not publicly available due to privacy or ethical restrictions.
